# Increasing translation of research evidence for optimal park design: a qualitative study with stakeholders

**DOI:** 10.1186/s12966-020-00952-5

**Published:** 2020-04-15

**Authors:** Jenny Veitch, Emily Denniss, Kylie Ball, Harriet Koorts, Benedicte Deforche, Anna Timperio

**Affiliations:** 1 Institute for Physical Activity and Nutrition (IPAN), School of Exercise and Nutrition Sciences, Deakin University, Geelong, Australia; 2 Department of Public Health and Primary Care, Faculty of Medicine and Health Sciences, Ghent University, C. Heymanslaan 10, 9000 Ghent, Belgium; 3 Physical Activity, Nutrition and Health Research Unit, Department of Movement and Sport Sciences, Faculty of Physical Education and Physical Therapy, Vrije Universiteit Brussel, Pleinlaan 2, 1050 Brussels, Belgium

**Keywords:** Park design, Stakeholders, Translation, Policy, Practice, Interviews

## Abstract

**Background:**

The design of parks is critical to ensure they are appealing, meet the needs of the community and optimise opportunities for physical activity, relaxation, exposure to nature and social interaction. There is currently a lack of understanding on how research evidence is informing park design and how to reduce the many challenges associated with research-practice-policy translation. Understanding how organisations use evidence for decision-making regarding park design is critical for reducing the research-practice-policy gap and ensuring evidence based strategies inform park design to support healthy active living. This study explored stakeholder perceptions regarding factors that influence the use of research evidence to inform park planning and design, and potential strategies to enhance effective translation of research evidence for optimal park design into policy and practice.

**Methods:**

One-on-one in-depth interviews were conducted with 23 stakeholders within the park design, planning and management sector. Participants shared experiences regarding: influences on park development and design; current park development policies; ways to facilitate use of evidence; and priorities for future research. Interviews were recorded and transcribed verbatim and content analysis performed using NVivo 12.

**Results:**

Research evidence is used and considered important for park planning; however, several barriers were highlighted: time and cost constraints; difficulties accessing research; and limited research relevant to specific needs. Developing partnerships between researchers and park developers and providing evidence in a more accessible format such as short summaries/reports, infographics, presentations, research updates and dedicated research databases emerged as key enablers. The main research gaps identified included research into park features to encourage visitation and cost-benefit analyses studies.

**Conclusions:**

This research is a first step to better understand strategies to promote more effective uptake and use of evidence to inform park planning. Researchers must develop multi-sectoral collaborations and generate policy-relevant research in a readily accessible, timely and user-friendly format to ensure evidence is used to enhance park design and ultimately public health.

## Background

Parks are important settings within cities that provide opportunities for physical activity, relaxation, exposure to nature and social interaction [1, 2]. However, they are not fully utilised, leaving scope for enhancing population health through increased park visitation. The design of amenities and features within parks is critical to ensure parks are appealing, meet the needs of the community and optimise visitation for all demographic groups [3]. Research evidence to inform optimal park design has been increasing in recent years [4] and natural experiments have shown park refurbishment to have a positive effect on park visitation and park-based physical activity [5–8]. However, there is a lack of understanding on how this evidence is informing park policy and planning, and how to reduce the many challenges associated with research-practice-policy translation.

Within built environment research, research translation challenges include a lack of alignment between research evidence generation and the needs of practitioners and policy-makers (e.g., absence of ‘policy-relevant research’) and a lack of understanding/training among researchers on ways to influence policy [9]. Strong evidence of the need for environmental change, in and of itself, may not be sufficient for enacting a practice or policy response. Critically, research evidence is only one aspect of the combined information used by policymakers to inform decision-making. In a survey of 152 UK policy-makers, 95% used local data to inform decision-making (80% perceived this as most useful) and the main sources of information were sourced from government websites and personal contacts; researchers were less likely to be consulted and research evidence was rarely seen as directly relevant to policy decisions [10].

Targeted knowledge translation strategies and support can; however, increase the use of evidence in policy and practice decision-making. In public health practice, promoting use of evidence in developing health promotion strategies tends to require multi-level and multi-strategy approaches. This may include, for example, facilitating practitioner/policymaker’s access to evidence, development of appraisal skills relevant to evidence integration, and strengthening of organisational cultures conducive to embedding evidence in practice [11]. Infrastructure to support translation activity within research settings may include more effective convening of relevant parties, better means of communicating evidence to change agents in a timely way, and incentives among researchers to pursue ‘strategic science’ (research designed to address gaps in knowledge important to policy decisions) [12].

In the health promotion field, engaging, collaborating and partnering with key stakeholders who will use and implement evidence is more likely to enhance the potential implementation of research in community settings [13]. Ideally such collaboration should occur during early conceptualization and research planning stages [13]. In active living research, which aims to influence the design of built environments (e.g., building of cities and parks) to increase population levels of physical activity, the following research translations/partnership strategies have been recommended: establishing networks between researchers, practitioners and policymakers; engaging knowledge brokers and advocates for more effective synthesis, communication and dissemination of evidence; and co-production of research with target stakeholders [9].

Despite the growing knowledge of the factors influencing translation of health promotion research into policy and practice, we are unaware of research that has specifically examined research-practice-policy translation of evidence for optimal park design. Little is known about what would help facilitate this translation, nor what format of research evidence would be most useful. Broadly speaking, we expect the barriers and facilitators to research translation to be consistent with those identified in previous evidence [9]; however, the pertinence of these issues among stakeholders responsible for park design specifically has not been investigated. Understanding how government and non-government community-based organisations use evidence regarding optimal park design to inform decision-making is critical to both reducing the research-practice/policy gap and maximizing the potential benefit of evidence-based strategies to improve environmental infrastructure to support healthy active living.

The aim of this study was to explore the perceptions of key stakeholders in park design, planning and management regarding: 1) factors that influence park development and design; 2) barriers and facilitators to using research evidence to inform park planning and design, and; 3) potential strategies to enhance effective translation of research evidence for optimal park design into practice and policy.

## Methods

This study was nested within ProjectPARK, a larger study examining the relative importance of park characteristics that attract visitors to parks and enhance park-based physical activity and social interactions among children, adolescents and older adults. This manuscript reports results from one-on-one interviews conducted with key stakeholders between May–July 2018.

Key practice and policy stakeholders (*n* = 51) in Australia were identified through various channels, including known contacts and collaborators (*n* = 20), attendees at relevant public forums and conferences (*n* = 24), and snow-ball recruiting (*n* = 7). The objective was to recruit participants holding a variety of positions and with varying expertise from both government and non-government organisations within the park design planning and management sector. Identified stakeholders received a plain language statement and an invitation to participate in a telephone or in-person interview via email. Non-responders received a follow-up email 2 weeks after the original email had been sent. Those who declined or did not respond to the follow-up email were not re-contacted.

Consenting stakeholders (*n* = 23, 45% of those contacted), participated in a semi-structured telephone (*n* = 21) or in-person (*n* = 2) interview with one of two researchers (JV or ED), at a time and location convenient to the participant. Seven participants were existing collaborators with the research team. Participants were asked to share experiences and opinions in response to the following six areas: 1) factors that influence park development and design; 2) current policies in place in their organisation in relation to park development or redevelopment; 3) ways to facilitate use of evidence to inform park design in their work or organisation; 4) barriers to using research evidence in policy and planning; 5) the most useful format for research evidence to be presented; and, 6) priorities for research evidence to support their work or organization. Participants also described their current position and experience in the field. Interviews lasted between 20 and 65 min and were audio recorded.

### Data analysis

The audio recordings were transcribed verbatim and imported into NVivo 12 (QSR International Pty Ltd., Melbourne, Australia) to manage coding of the data. Data was then analysed using a summative content analysis approach [14]. All interviews were read carefully, followed by inductive coding into frequently recurring subcategories and groups based on the survey questions. A coding protocol was developed prior to analysis; however, this was amended throughout the coding process based on the interview data. The assignment of the subcategories and grouping into categories was performed by two researchers (JV and ED) and any clarification or disagreements were discussed until consensus was reached.

## Results

Overall, 23 stakeholders were interviewed (65% male). They represented companies located in urban and regional areas of Victoria and Queensland, Australia. Eighteen were employees within government organisations (e.g. local councils, Department of Environment, Land, Water and Planning, Parks Victoria, Water Authority) and five were employed in private companies (e.g. playground design, landscaping, leisure facility management). Most stakeholders (*n* = 14) held planning and management roles (e.g. Manager of Urban Design, Strategic Planning, Recreation Planner, Open Space Co-ordinator, Liveability Project Officer), four held roles in design (e.g. Landscape Architect) and five held roles that combined planning, management and design. Nine stakeholders were involved in policy development. The average length of time in their current position was 7 years and 16 participants had more than 10 years’ experience in park design/management. A summary of the research findings discussed below are presented in Table 1.

**Table 1:** Summary of key findings

**Factors influencing decisions to (re) develop parks and park design:**	
• Overarching policy and strategic planning documents	
• Condition of existing parks	
• Availability of land and characteristics of site	
• Desire to meet evolving needs of the community	
**Current use of evidence in policy and planning:**	
• Evidence currently used included: academic evidence, evidence collected by their own organisations and evidence distributed by other non-academic organisations	
**Barriers to using evidence to inform park design:**	
• Time and cost constraints: enhanced due to deadlines and short time frames	
• Difficulties locating and accessing relevant research: enhanced due to lack of skills and knowledge of how to find relevant research and insufficient access to databases/academic literature	
• Lack of relevant research relevant to real-world settings	
**Ways to enhance evidence-based park design:**	
• Develop research partnerships between research academics/institutions and park developers/planners	
• Provide more accessible research, communicated in a timely, succinct and informative format such as short summary reports, infographics, regular research updates, dedicated research databases, and conference and seminar presentations	
**Research gaps:**	
• Identifying park features that would encourage visitation across varying demographic groups to inform the planning and design of future park developments and re-developments	
• Cost-benefit analysis studies of park and open space developments	

### Factors that influence the decision to (re) develop parks and park design

Overarching policy and strategic planning documents that outline priorities for developing new parks and redeveloping existing parks were reported as the key factor that influenced park (re)development. Forty-three percent of participants’ organisations had current policies in place in relation to park (re)development. A further 9% worked for organisations that did not have their own policies but were contracted by councils that did.

*“We have our local government infrastructure plan which sets out in the next 20 years which parks we’re going to develop, how much it’s going to cost. That’s based on population modelling, needs, areas with specific needs or growth.” (Parks and Natural Areas Planner)*

*“So there’s generally a policy position that will underpin the decisions to provide parks in the first instance.” (Sports and Recreation Planner)*

The condition of existing parks and their maintenance and upgrade requirements were reported to influence decisions regarding park development. Parks that had not been updated within a certain timeframe or had features that required maintenance were prioritised for redevelopment.

*“So, when we’ve got parks reaching the end of their useful life we look at what opportunities exist to replace and upgrade them. We’ve got asset management plans, and you get a pretty good gauge on the equipment. We do annual audits on our parks and playgrounds which identify what meets standards and what doesn’t.” (Open Space Coordinator)*

Availability of land in both established and developing neighbourhoods were reported as key influences on park (re)development. Characteristics of the site, such as size, landscape, climate and wildlife were important factors that influenced park design.

*“So often we deal with issues, like particularly in new areas of growth, how much open space should be provided. How it’s provided can have an effect on whether it’s used or not.” (Manager for Planning Projects)*

*“We explore what opportunities we have to leverage the natural conditions and advantages of the landscape to ensure that we reinforce or in some cases recreate a local sense of identity and character.” (Senior Strategic Planner)*

Endeavoring to meet the evolving needs of the community was a major influence on park (re) development and design. Community engagement was commonly used as a tool to evaluate the community’s needs and inform park design.

*“We might look at an upgrade if we don't feel the park meets the needs of the current community, so might be somewhere like x which was basically a rural park when it was formed in the 1980's and now it has houses all around the boundary, … … so we need to look at what facilities we are providing, are there trail links to the right places and are there toilets in the right places and have we got the mix of activities right.” (Open Space Planner)*

*“We’re very much driven by community kind of wants and needs, and through consultation. So if we’re planning a space, we’ll go out to the community and say, “What would you like to see here?” kind of how they use the space, what they like about it, what they’d like to see changed, and then we’ll try and incorporate all of that into a plan.” (Coordinator of Open Space)*

### Current use of research evidence in policy and planning

All participants reported using at least one form of research evidence to inform park policy and/or planning. Stakeholders reported using academic evidence, commissioning their own research, and using research findings distributed by non-academic organisations including national data.

#### Academic evidence

Participants generally regarded research evidence as important. They recognised the benefits of using evidence to inform park design and policy and the value of establishing partnerships with academics and research institutions.

*“On a lot of our policy development work we test it and we look to research to justify the position. For example, one of the things that we’ve recently done where we’ve worked with universities is looking at what’s reasonable access to different things, including open space … and the policy guidance has been developed in conjunction with the research.” (Manager for Planning Projects)*

*“ … . it also comes from published research that we will seek out and access, and my conversations with [researchers] are about us getting more specific and looking at issues and working with institutions to really delve into I suppose those more challenging or unknown issues that we are looking to create solutions for.” (Managing Director)*

#### Research conducted by organisations

Stakeholders also reported collecting their own evidence to inform park planning and development. This typically included surveys and interviews with park users, pedestrian counters to record park use, observational techniques and project evaluation.

*“We commission a bit of research ourselves, you know around our existing spaces. So, we get people in to do footpath counts and survey visitors to determine their experience.” (Green Infrastructure Support Officer)*

*“ … we do have a lot of pedestrian counters, which are on our shared trails, measuring usage. We've been doing a lot of upgrades so we can monitor the success of those upgrades.” (Coordinator of Public Space Design)*

#### Research conducted and/or distributed by other non-academic organisations

Stakeholders also reported using evidence filtered through from peak bodies (i.e. organisations that represent the interests of the industry as a whole) or other organisations.

*“We engage a lot of landscape architects. So, we back their judgment and their understanding of design issues and contemporary or current research on those things. Our peak body is Parks and Leisure Australia … … .. so we often tap into that kind of resource. But yeah, we don’t have any relationships with universities, schools, or anything, so we’re left to our own devices.” (Open Space Co-ordinator)*

*“We don’t really have all that much time to sift through journals and stuff, so it’s more we rely on those sort of peak bodies that we’re involved in to send us information and reading articles and the papers through them.” (Coordinator of Passive Reserves)*

### Barriers to using evidence to inform park design

Despite the acknowledged benefits of using evidence to inform park planning, multiple barriers to using evidence were discussed. The four main barriers that emerged included time constraints, difficulty locating and accessing research, cost, and relevance to real-world settings.

#### Time constraints

Participants reported time constraints as a barrier to using research evidence to inform policy development and park design. Deadlines and short time frames made it more challenging for stakeholders to conduct research or utilise existing research.

*“One of the biggest challenges is the time frame. We’ve often got a lot of pressures to deliver things, at a certain time frame, often short time frames. Even when we are doing the policy development it has tended to be quite quick. Whereas research often takes a lot longer and feeding in the exact information we need might not be able to be done within the time frame. ....... we operate on a very short cycle and with any policy guidelines there is a real tension between trying to create quality to actually and to deliver on something.” (Manager for Planning Projects)*

#### Locating and accessing research

Difficulty finding and accessing relevant research emerged as a challenge for most stakeholders. Participants reported having limited skills to search for and locate the most relevant research and limited knowledge of what is available. Insufficient access to databases and academic literature within their organisations was also highlighted as a barrier. Time constraints exacerbated the challenge of locating and accessing relevant research.

*“It’s a job trying to pinpoint where in the research community, where to go and who is doing what. You end up working with one and find out it’s actually been done somewhere else as well or slightly different. So, that can be a real challenge actually, like you sort of feel like you are going through the right channels and getting the right research and then you might actually find that ah, there’s someone over here doing something that’s even more relevant, and you’ve already started to working with somebody else.” (Manager for Planning Projects)*

*“In some cases there’s a requirement for subscriptions and payments and things like that and we’re usually not in a position to pursue research evidence and information that way, so we’re really almost always relying on kind of open source information.” (Senior Strategic Planner)*

*“..so sometimes we don't have the time, but I think quite often people don't know where to look or what sort of questions would be useful to ask.” (Open Space Planner)*

#### Cost

Stakeholders described insufficient funding as a barrier to undertaking or using research evidence.

*“It’d be great to have a researcher or someone who can research beyond my capabilities, that’s the main thing but of course. you know everything’s cost prohibitive.” (Director/Landscape Architect)*

*“It’s difficult as a consultant, because if you’re not paid to do something it’s very difficult to generate that research, because we’re obviously a commercial entity so we’re trying to make ends meet.” (Director/Landscape Architect)*

#### Relevance of research to real-world settings

A lack of research relevant to the needs of practitioners was reported as a barrier to incorporating evidence into park policy and design. Respondents indicated that the particular research evidence they required was often unavailable.

*“I think relevance is probably just one of the main issues, and I don’t know, from a research point of view maybe the relevance can be quite difficult to establish as well in the real-world setting. Hopefully your leaders as well know what the industry wants, and then we should be vocal as well, and saying, well this is actually what we’d like; we don’t have data on blah, and if that can work, that would be brilliant.” (Senior Project Coordinator)*

### Ways to enhance evidence-based park design

While the majority of participants used academic evidence, about one-third indicated that it could be used more often and more effectively. When asked what would make it easier to use research evidence, research partnerships and more accessible evidence emerged as key enablers.

*“It’s still an area I think that’s a bit of a gap for us a lot of the time … Yeah, it’s probably one of the areas that doesn’t seem to be as readily accessible. We’ll access it where it’s available, but we’ve been talking as a directorate about how we can make better use of our university research.” (Manager Visitor Planning)*

#### Research partnerships

The majority of stakeholders proposed that partnerships between park developers, academics and research institutions would help facilitate the use of research evidence to inform park policy and design and also help ensure the research meets their needs.

*“I think if we’re able to partner with institutions such as yours and collaborate on what sort of things you want to research and what sort of things we might want to research, I think there’s definitely a lot more that could be done in that space.” (Senior Project Coordinator)*

*“So we need to kind of have the relationships first … not with individuals necessarily, but with, I guess, faculties so that we can then not only share information backwards and forwards a bit better, but we can actually work and plan those sort of research projects more collaboratively so that if we’ve got particular research needs then the universities and the students can be undertaking research that’s very targeted to a real-world problem.” (Manager Visitor Planning)*

*“Well, on a project it’d be great to have a researcher or someone who can research beyond my capabilities.” (Director/Landscape Architect)*

#### More accessible research

Making research more accessible was mentioned by stakeholders as an enabler for using evidence to inform policy and design. Stakeholders preferred research to be delivered in formats that enabled key findings to be communicated in a timely and informative fashion.

Short research summaries and reports were suggested by most stakeholders as an ideal format for communicating research evidence. There was a strong preference for a succinct delivery of the key research outcomes.

*“So if it was coming to us in a very accessible format, then we would be far more inclined to be incorporating it.” (Open Space Coordinator)*

*“Look because of time constraints probably an executive summary is sufficient for you to know whether you want to read further I suppose or link to something that gives you greater depth of detail.” (Director/Landscape Architect)*

The visual and simplified presentation of information via an infographic was mentioned as a useful format of evidence by stakeholders.

*“The simple, hard data, and infographics to me is the best way to sell something. It gets my attention straight away. I digest the information just on an A4 page, I realise what this is about, comprehend what’s this project about and what have we learned from this.” (Livability Project Officer)*

A regularly disseminated park research update was suggested by about one-third of respondents as a means to make research more accessible to park developers.

*“If I got an email once every week or two about a new research paper on research into parks and what works well or whatever, that would be fantastic. It would save me doing that research.” (Parks and Natural Areas Planner)*

In-person, verbal delivery of research evidence through conference and seminar presentations and small group discussions were mentioned by almost one-third of respondents as a convenient format for information exchange.

*“I think that certainly conferences and meeting with the people who are putting the research together and interpreting it, and having conversations about what it means, I think having those one on ones, or small group sessions to delve more deeply, would be really valuable.” (Managing Director)*

A research database dedicated solely to park related research to improve access was also recommended by several stakeholders.

*“So if there was like a portal or a hub … if there’s some kind of place that has a centralised area where you could have say different headings or whatever of research about for example the climate change street trees. Then there could be links to documents or listed within that so you could very quickly find what’s been done and what location that’s in to see if it’s relevant.” (Director/Landscape Architect)*

### Research gaps

Two main research gaps were identified by stakeholders. Firstly, stakeholders called for research into current and emerging park features that would encourage visitation across varying demographic groups to inform the planning and design of future park developments and re-developments.

*“I think it would likely be what makes, what encourages people to use open space … more sort of a sophisticated approach which I think councils obviously do more than we do, but would be really useful.” (Manager for Planning Projects)*

*“It’s really good to get to know what demographically people want to see in their parks as well. So, getting that information, having it up-to-date.” (Senior Project Coordinator)*

Secondly, cost-benefit analysis studies of parks and open spaces was recommended by stakeholders as an important research area. Respondents identified the necessity for this when applying for funding and justifying spending on park (re)development.

*“I think cost analysis would be really interesting, particularly because we find it harder to justify some of the things we do in terms of cost benefit analysis.” (Coordinator of Public Space Design)*

*“So unless we really have a good handle on known visits, existing visitation, projected visitation, actual spend and economic benefit to local regional communities, it’s much harder to convince funding bodies to actually give us that money.” (Manager Visitor Planning)*

## Discussion

Key informant interviews were conducted to better understand factors influencing park development and design and how to facilitate enhanced knowledge exchange and transfer between researchers and decision-makers relating to park design. This research is a first step to better understand potential strategies to promote more effective uptake and use of research evidence to inform park planning and design. Creating cities and active environments that facilitate physical activity is a global priority and a key strategic objective of United Nation’s 2030 Agenda for Sustainable Development [15] and the World Health Organisation’s Global Action Plan on Physical Activity 2018–2030 [16]. Ensuring our parks are designed to meet the needs of our community and maximize visits by all people is critical for public health.

Consistent with a study of 23 policy makers in the UK [17], the stakeholders generally appeared to hold academic evidence in favourable regard and either already used it, or would like to use research evidence more often. However, several barriers to using and accessing evidence were highlighted. These included time constraints, cost, difficulty locating and accessing relevant research and lack of personnel to search for evidence, and limited research that is relevant to their specific needs.

To help overcome these barriers, participants suggested that more accessible evidence communicated in a timely and informative fashion such as short summaries and reports, infographics and conferences or seminar presentations, regularly disseminated park research updates and dedicated research databases would be helpful. These are potentially feasible strategies that need to be tested and developed with a range of stakeholders who require evidence in varying formats to ensure it meets their needs. There is limited evidence exploring the uptake of evidence-based resources to inform practice from the practitioner’s perspective [18]. However, previous research suggests that presenting research evidence to policy makers and practitioners through multiple channels and formats in an easy-to-understand, short and varied format is crucial to receptivity [19]. It is also important to acknowledge that for many researchers, effectively translating research findings in a format so evidence can reach and inform practice is a challenge and the needs of practitioner’s are often not well understood by researchers [20].

Participants also spoke about the importance of developing partnerships between researchers and industry. Establishing partnerships between park developers, academics and research institutions and involving stakeholders in research is mutually beneficial and has many potential advantages. For example, it has been suggested that natural experiments are more likely to influence policy and practice when undertaken from the outset in partnership between researchers, policymakers and practitioners [21, 22]. This has successfully occurred in some recent natural experiment studies evaluating park refurbishment in Australia [23, 24] and in the US [25]; however, future research needs to explore mechanisms to more routinely and strategically involve park developers and planners in the research study from inception. It has also been previously recognised that two-way communication between academics and stakeholders and inviting community input helps to ensure research addresses local issues [19].

Stakeholders identified that more comprehensive and up to date evidence on park features to encourage visitation is required to help inform future park planning and development, and cost-benefit analyses studies are of enormous value when seeking financial investment for park (re)development. Cost-benefit analysis plays a key role in decision-making; however, there is limited international evidence on the cost-benefit of upgrades to existing parks or the development of new parks for visitation, physical activity or other health outcomes such as mental health and well-being [26–28]. A recent study in Melbourne, Australia showed that a new play-scape installation in a metropolitan park located in an area of socioeconomic disadvantage was cost-effective in terms of increasing physical activity [29]; however, future cost-benefit analysis studies are required in parks of varying size, amenity and location and in neighbourhoods of varying socio-economic status. Considering the restricted availability of funding and competing priorities, cost-effectiveness evidence is critical.

Overall, the findings from this research highlight that multi-sectoral collaborations between relevant fields is essential to enhance park design, park visitation and ultimately public health. It is critical that researchers and practitioners work together to optimise outcomes and fulfill needs across disciplines. Future research needs to examine how these partnerships can be established and maintained successfully. Although this research examined knowledge exchange and transfer between researchers and decision-makers relating to park design, it is important to acknowledge that the strategies and lessons learnt could be applied to other fields of research and topic areas.

### Strengths and limitations

This research study provided an in-depth exploration of stakeholders’ views. Participants included stakeholders with wide ranging roles and experience with varying investment in park design, which provided a diversity of perspectives. All interviews were conducted by the same two researchers and transcription of interviews was outsourced to a transcriber external to the research team. Qualitative research has been acknowledged as an important way of conducting meaningful inquiry to bridge research, policy and practice [19]. Limitations included the recruitment strategy which involved targeting known contacts/collaborators and snowball sampling which may have resulted in recruitment of participants with shared experiences. It is therefore possible that there was an over-representation of certain perspectives. Known collaborators and contacts may have also felt “pressure” to provide socially desirable responses; however, this was also a strength as it made it possible to specifically target representation across different types of organisations to ensure key people were involved. Snowball recruitment also led to the recruitment of relevant and experienced participants. It is important to acknowledge; however, that it is possible that stakeholders with differing views and perspectives to those presented in this study were not included. It is also possible that participants provided socially desirable responses as research staff were interviewing them. Finally, all stakeholders were employed within an Australian context so the findings may not be generalisable to other countries, in particular low or middle-income settings.

## Conclusion

Research evidence is used by and considered important and relevant to park planners and designers. However, there are many challenges associated with using evidence to inform park policy and planning. Researchers must generate policy-relevant research in a readily accessible, timely and user-friendly format. Further research is needed to better understand how to successfully establish and maintain relationships/partnerships between researchers and key stakeholders and how to facilitate increased use of research evidence in park policy and design.

## Data Availability

The dataset used and/or analysed during the current study are available from the corresponding author on reasonable request.
